# Acute Asthma in the Pediatric Emergency Department: Infections Are the Main Triggers of Exacerbations

**DOI:** 10.1155/2017/9687061

**Published:** 2017-10-12

**Authors:** Arianna Dondi, Elisabetta Calamelli, Valentina Piccinno, Giampaolo Ricci, Ilaria Corsini, Carlotta Biagi, Marcello Lanari

**Affiliations:** ^1^Pediatric Emergency Unit, S. Orsola-Malpighi Hospital, Department of Medical and Surgical Sciences, University of Bologna, Bologna, Italy; ^2^Pediatric and Neonatology Unit, Imola Hospital, Imola, Bologna, Italy; ^3^Pediatric Unit, S. Orsola-Malpighi Hospital, Department of Medical and Surgical Sciences, University of Bologna, Bologna, Italy

## Abstract

**Background:**

Asthma exacerbations are a common reason for Emergency Department (ED) visits in children.

**Aim:**

To analyze differences among age groups in terms of triggering factors and seasonality and to identify those with higher risk of severe exacerbations.

**Methods:**

We retrospectively revised the files of children admitted for acute asthma in 2016 in our Pediatric ED.

**Results:**

Visits for acute asthma were 603/23197 (2.6%). 76% of the patients were <6 years old and 24% ≥6. Infections were the main trigger of exacerbations in both groups; 33% of the school-aged children had a triggering allergic condition (versus 3% in <6 years; *p* < .01). 191 patients had a previous history of asthma; among them, 95 were ≥6 years, 67% of whom were not using any controller medication, showing a higher risk of a moderate-to-severe exacerbation than those under long-term therapy (*p* < .01). Exacerbations peaked in autumn and winter in preschoolers and in spring and early autumn in the school-aged children.

**Conclusions:**

Infections are the main trigger of acute asthma in children of any age, followed by allergy in the school-aged children. Efforts for an improved management of patients affected by chronic asthma might go through individualized action plans and possibly vaccinations and allergen-avoidance measures.

## 1. Introduction

Asthma is the most common chronic disease in childhood. In Italy, it has been estimated that up to 9.5% of children aged 6-7 years and 10.4% of teenagers aged 13-14 years have ever been affected by this condition [1]. Acute asthma exacerbations are one of the most common reasons for presentation to the Emergency Department (ED) and for hospitalization in the pediatric age. In the United States, among asthmatic children nearly 60% have one or more acute exacerbations each year [2, 3], and up to 20% require ED visits annually [4]. Moreover, asthmatic patients treated in the ED are at higher risk for future exacerbations and any single severe acute episode may progress to life-threatening respiratory failure [2, 5].

Pediatric asthma has different patterns according to the children's age. In the preschoolers (0–5 years old), acute wheeze is often induced by infections of the lower airways, whereas in the school-aged children (6 years and older) it usually signifies underlying asthma [6] and allergy. These differences account for specific seasonal patterns in the number of ED visits for asthma. In the temperate latitudes of the Northern Hemisphere, a peak in exacerbations is described in the month of September: this well-known entity is called the “September asthma epidemics,” coincides with the start of the school year, and is likely due to a combination of infectious, allergic, environmental, and climatic triggers [7]. Understanding the seasonal variations of ED visits due to asthma may have important therapeutic implications in terms of a proactive treatment of at-risk subjects.

We performed a retrospective analysis of all the Pediatric ED visits for asthma in the past year in our hospital to analyze the differences among age groups in terms of triggering factors and seasonality and to identify if there are any patients' groups which are more at risk of severe exacerbations and hospitalization.

## 2. Methods

We retrospectively revised all the files of children aged 0–14 years who were visited for acute asthma from 1 January to 31 December 2016 in the Pediatric ED of S. Orsola-Malpighi University Hospital of Bologna, Italy. All the patients with a diagnosis of “acute asthma,” “wheezing bronchitis,” and “bronchospasm” were included.

Our center is an urban, academic, tertiary care Pediatric Emergency Unit, consisting of a Pediatric ED with approximately 23000 visits per year of children aged 0–14, a short-stay observation with 6 beds, and a ward with 28 beds. Patients arriving at the ED are registered by a triage-qualified nurse and seen first by pediatric residents and the attending physician. Two more nurses are available for the ED patients and 1 for the short-stay observation.

Triage acuity is defined by a 4-grade colour scale, white being the less urgent and red being the most. A white code means that the condition is not relevant or does not have an acute onset and is not affecting the vital signs; it can be reevaluated by the triage nurse if the symptoms modify and it might gain a higher priority. A green code indicates patients with normal vital signs but relevant, acute-onset symptoms; they should be reevaluated by the triage nurse every 30–60 minutes until medical evaluation. A yellow code is for those with severe lesions and altered vital signs that require rapid medical evaluation, so that these cases are closely communicated to the attending physician. A red code means that the condition is life-threatening (vital functions are compromised) and the patient requires immediate evaluation.

A Pediatric Intensive Care Unit is in the same building and intensivists' consultancies are available as needed.

For each patient presenting to the Pediatric ED, a record form is filled in by the nurse and medical staff, including demographic data, anamnestic information, vital signs, physical examination, treatment performed in the ER (if done), clinical reevaluation after treatment, diagnosis, discharge modality, and home therapy prescription when applicable. For children requiring a short-stay observation, prescribed examinations, therapy, and subsequent reevaluations are also noted on the same form.

The etiology of asthma exacerbations was considered as infectious if the episodes were concomitant to a respiratory tract illness documented by the clinical examination and/or laboratory or radiologic investigations; as allergic when there had been a clear exposition to a likely triggering allergen in a susceptible individual (known atopic or with a family history of allergic disease or with previous asthma episodes) and with no concomitant respiratory infection; as exercise-induced when the exacerbation had been precipitated by physical activity; if no overt cause could be ascertained, the etiology was classified as “unknown.”

The following data were considered for the study: age and sex of the patient, month of the visit, triage colour tag, acute asthma severity (see Table 1) [8], etiology of the exacerbation, previous history of asthma or other chronic conditions (if reported), use of long-term control therapy for asthma, vital signs, presence and severity of dyspnea, severity of the exacerbation, administered therapy, and discharge modality (home, short-stay observation in the ED, and admission to the Pediatric Unit Ward or to the Pediatric Intensive Care Unit).

The data were collected in a Microsoft Excel® database. A descriptive analysis was performed for continuous variables (median, 25th and 75th percentiles). Data distribution was checked using MedCal Statistical Software (Version 17.4 MedCalc Software, Ostend, Belgium).

The associations between qualitative variables were evaluated with Chi-square test. Results were deemed as significant for *p* < .05. STATA 7.0® (Stata Corporation, 4905 Lakeway Drive, College Station, Texas 77845, USA) was used for the analysis.

## 3. Results

In 2016, our Pediatric ED registered 23197 visits, 603 (2.6%) of whom for “acute asthma,” “wheezing bronchitis,” or “bronchospasm”. Of the 603 patients, 491 (81%) were discharged, while 112 children (19%) were admitted, 15% (*N* = 89) to a short-stay observation (up to 36 hours) and 4% (*N* = 23) to our Pediatric Unit Ward; 1 child was admitted to the Pediatric Intensive Care Unit after initial treatment in the Pediatric Ward.

The main features of the study population are described in Table 2. Sixty-five percent of children (*n* = 394) were males, while the rest of them (*n* = 209; 35%) were females. The median age of the patients was 3.1 years (range: 2 months–14 years). Forty hundred fifty-nine patients were younger than 6 years (median 2.1 years; *n* = 206 < 2 years; *n* = 253 ≥ 2 years and <6 years), while the rest of them (*n* = 144; 24%) were school-aged children and adolescents (median 8.7 years).

**Figure 1:**
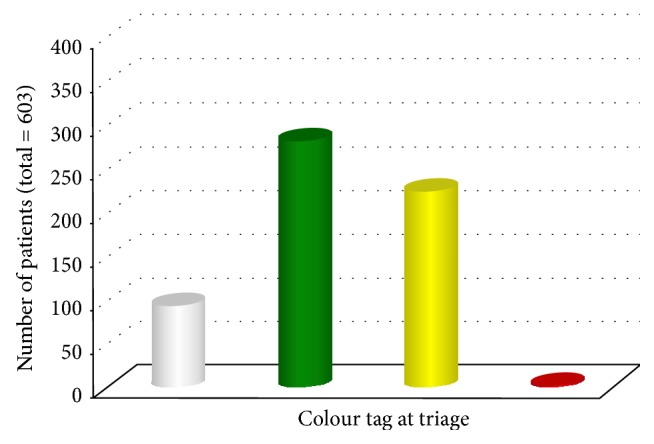
Colour tag at triage of 603 patients aged 0–14 years who were visited for acute asthma from 1 January to 31 December 2016 in the Pediatric ED of S. Orsola-Malpighi University Hospital of Bologna.

At triage, 94 patients (15.6%) were given white colour tag, 282 (46.8%) green, 225 (37.3%) yellow, and 2 (0.3%) red (Figure 1). The peripheral oxygen saturation (SpO2) upon arrival was <92% in 5.3% of cases, 92–95% in 34.4%, and >95% in 60.2%. The severity of the episode was classified as mild in 340 cases (56%), moderate in 237 (39%), and severe in 26 (4%), while none of the episodes was classified as life-threatening.

**Figure 2:**
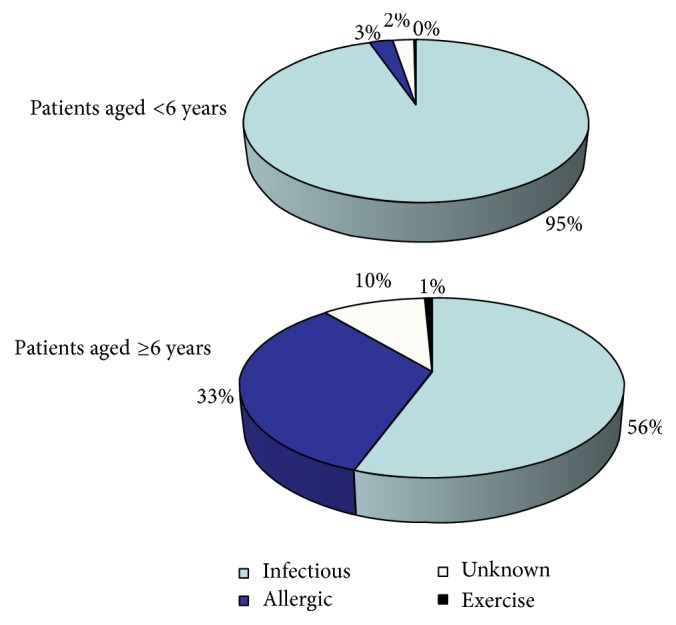
Etiologies of asthma exacerbations in 603 patients aged 0–14 years who were visited for acute asthma in 2016 in the Pediatric ED of the Pediatric Department of S. Orsola-Malpighi University Hospital of Bologna and divided into 2 subgroups: patients younger than 6 years (*n* = 459; 76%) and those aged 6 years and older (*n* = 144; 24%).

The etiologies of the exacerbations are described in Figure 2. Most of the episodes occurring in children <6 years had infectious etiology (95% versus 56% in children ≥6 years; *p* < .01), while in children ≥6 years 33% of the exacerbations were triggered by an allergic condition (versus 3% in children <6 years; *p* < .01).

**Figure 3:**
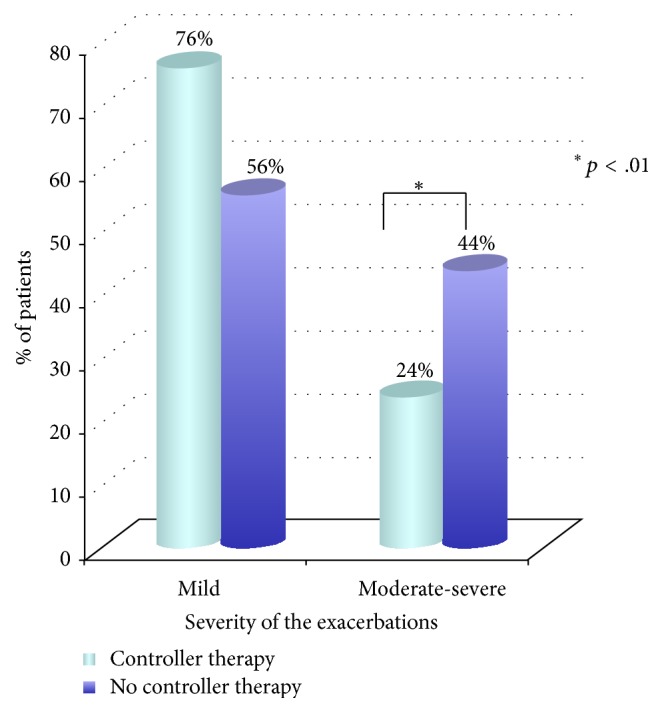
The severity of the exacerbations in patients ≥6 years with a previous diagnosis of asthma (*n* = 95) who were visited for acute asthma in 2016 in the Pediatric ED of the Pediatric Department of S. Orsola-Malpighi University Hospital of Bologna and divided into two subgroups (“controller therapy” and “no controller therapy”).

One hundred ninety-one patients (31%) had a previous history of asthma or wheezing bronchitis and 54 of them (28%) were using an asthma controller therapy. Most had a mild exacerbation (*n* = 110; 57%) and 69 (36%) moderate, while only 12 (6%) presented a severe asthma attack. Sixteen percent of them required admission either to the short-stay observation or to the Pediatric Ward. Among the children with a previous asthma diagnosis, 95 (49%) were ≥6 years and 63 of them (67%) were not using any controller therapy (either for no prescription or for no use). The severity of the exacerbations in patients who are ≥6 years with a previous diagnosis of asthma and divided into two subgroups (controller therapy, no controller therapy) is shown in Figure 3: asthmatic patients not using a controller medication were more likely to have a moderate-to-severe exacerbation than their peers under long-term therapy (*p* < .01).

**Figure 4:**
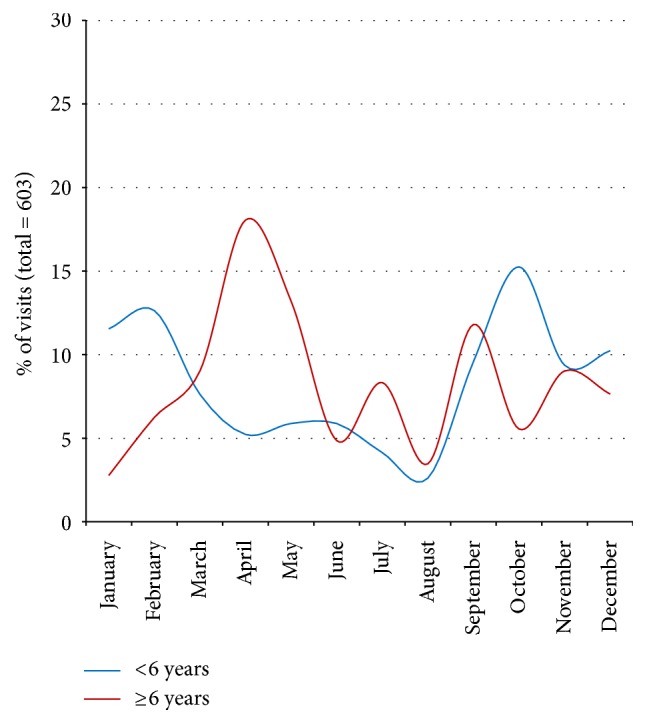
The seasonal trend of the asthmatic exacerbations in 603 patients who were visited for acute asthma in 2016 in the Pediatric ED of the Pediatric Department of S. Orsola-Malpighi University Hospital of Bologna and divided into 2 subgroups: patients younger than 6 years (*n* = 459; 76%) and those aged 6 years and older (*n* = 144; 24%).

The seasonal trend of the exacerbations is shown in Figure 4. Children <6 years showed a peak of exacerbations in autumn and winter concurrently with the infectious epidemic season, while school-aged children and adolescents showed a different trend with a peak in early autumn and another one during the pollen season.

## 4. Discussion

The present paper analyzes 603 consecutive visits for asthma exacerbations in our Pediatric ED. The proportion of visits for asthma that we describe (2.6%) is similar to that reported by Nath and Hsia in 2015 in a United States study (2.8%) [9]. According to what is described by Bekmezian et al. [10], most patients were males and preschool-aged children. “Green” and “yellow” triage priority labels were the most frequently assigned to our patients; the severity was most often judged as mild or moderate and only in 4% of the patients as severe. Bekmezian et al. studied 1249 Pediatric ED visits for moderate-to-severe asthma exacerbations and reported that only 1% of them had a triage priority code “1” (comparable to our “red”); in most cases, a “2” or “3” code was assigned and, less frequently, a “4 or 5” code [10].

### 4.1. Etiology of Asthma Exacerbations

Our data show that respiratory tract infections are the most common trigger for asthma exacerbations in both preschool- and school-aged children (Figure 2). The importance of respiratory infectious agents, both viruses (e.g., respiratory syncytial virus, rhinovirus, metapneumovirus, parainfluenza virus, and coronavirus) and bacteria (e.g.,* Chlamydia pneumoniae, Mycoplasma pneumoniae*), in the development of asthma exacerbations has been stressed by several authors [11–14]. The role of infections in acute wheezing is even more evident in the first years of life. Indeed, our results highlight that almost all the wheezing preschool-aged children had an underlying respiratory illness.

On the other hand, one-third of the children aged 6 or more had an asthma exacerbation induced by an allergic trigger, whereas in 10% the etiology could not be made out during the ED visit. In fact, several agents can elicit acute asthma: respiratory tract infections, environmental allergens, pollutants, and stress [14, 15]. Moreover, synergistic interactions between these factors might also very likely play a role [14].

### 4.2. Seasonality of Asthma Exacerbations

The existence of seasonal cycles of asthma exacerbations is well established [7, 14, 16–18]. Children frequently experience a worsening of asthma when they return to school after the summer break; the peak of this “September asthma epidemics” is around 2 weeks after the start of school [7, 14, 16]. In 2006, Johnston and Sears examined data from the Canadian Institute of Health Information and found a clear seasonal pattern in ED visits and hospitalizations for asthma, showing, for the 5–15 years' age group, a relatively stable trend for the first 6 months of the year, a trough in the summer months, and then a rapid and substantial increase in mid-August, reaching the peak 2 weeks after return to school; in children aged 2–4, the pattern was similar but the peak less striking [16]. More recently, Cohen et al. analyzed data from 82234 asthmatic children and found that unscheduled primary care physicians visits and drug prescriptions for asthma were fluctuating during the first part of the year, had a decline in the summer months, and peaked in September with a lower peak in autumn [7].

Similarly to what is reported in the literature, our data show an increase in ED visits for asthma in early autumn, but with a difference between children aged 0–5 and ≥6 years (Figure 4). In the younger group, the peak starts in September, reaches its maximum in October, and then continues for the whole autumn and early winter. In the school-aged children, there is a peak only in September which is, however, lower than that observed during the spring, mainly in April and May.

The diversity between the two age groups can be explained by the different triggers that can induce asthma exacerbations, particularly allergic factors that hardly play a role in the preschool-aged children but are important in the school-aged ones. However, the reasons for the discrepancy between our data and those published in the literature, specifically the spring peak that we clearly highlight, are unclear. Possibly, differences in the climate and in the grass pollen spring outbreak might be part of the explanation.

### 4.3. Exacerbations in Asthmatic Patients

Understanding the proportion of children with a preexisting diagnosis of asthma seeking advice in the ED for an exacerbation is important both for the individual patients and for the community, in terms of disease control and quality of life, and of healthcare costs, respectively. A history of acute asthma crises in the previous season or year was shown to be a risk factor for new exacerbations in 400 asthmatic patients aged 6–20 years [19]. Acute asthma exacerbations are largely preventable [2], and so are ED visits and hospitalizations for this reason [9, 20–22]. In the United States, there have been federally funded initiatives to reduce this burden, such as school-based programs and a widespread dissemination of the guidelines, but a study performed on the total Pediatric ED visits from 2001 to 2010 highlighted that, despite these measures, the overall rate of potentially preventable ED visits for asthma did not change significantly over time [9].

Among our patients, almost one-third had a previous diagnosis of asthma or wheezing. Half of them were in the school age, meaning that they were the real asthmatics of the case series, according to what is previously described by Stein and Martinez [23]. For them, the chance of needing hospitalization was higher than their peers with no previous asthma diagnosis. Among this group of older children with known asthma, up to 2/3 were not using controller medications, either for no prescription or for low compliance, and, according to our results, were at higher risk of a moderate-severe exacerbation than those using it. Controller medications are indicated in children with persistent asthma, and their correct use has been shown to reduce exacerbations and thereby ED visits for asthma [21, 24]. Evaluating asthma severity was beyond the goals of our study; thereby we cannot say how many of the 63 asthmatic children aged over 5 years and not using long-term controller therapy would have had a real need of such a prescription. However, the fact that so many asthmatic children were not using controller therapy, although our National Health System provides a pediatrics-specialized general practitioner for all children until the age of 14, seems to point out the need for an improvement in the territorial network of asthma management.

It is well established that patients with an acute asthma exacerbation are at high risk for future episodes [2]. This specific patient group should receive appropriate controller therapy, but leaning on following outpatients' visits has been demonstrated not to be reliable, as shown by a study on 3435 ED visits for asthma in children, only 18% of whom had a prescription for inhaled corticosteroids in the 2 months following the ED visit, only 12% attended follow-up appointments, and only 5.2% received both [25]. Even if the guidelines do not state that ED physicians should identify children with persistent asthma and start long-term therapy, some authors suggest that, in appropriate patients, inhaled corticosteroids might be prescribed at ED discharge [24, 26]; however, the rates of such prescriptions are low [25, 26].

As confirmed by our results, infections and allergy are the most frequent triggers in asthma exacerbations. In the light of this, also preventive measures are confirmed to play a relevant role in the management of pediatric asthma and should be strongly recommended in these patients. The efficacy of influenza inactivated vaccine in terms of reduction of the episodes of acute respiratory tract illnesses, asthma exacerbations, hospitalizations, and acute pharmacotherapy use in pediatric patients with mild persistent has been suggested [27]. In addition to this, the Advisory Committee on Immunization Practices has extended the recommendation also for pneumococcal vaccination to asthmatic children and adolescents aged 6–18 years [28].

Moreover, specific allergen-avoidance measures should also be addressed to all sensitized patients, for example, removing furry animals from the house in case of pet dander allergy, encasing mattresses in allergen-impermeable covers to reduce house dust mites exposure, cleaning from surfaces indoor molds using bleach solutions, and reducing pollen indoor exposure by closing windows and doors during high pollen counts in pollen allergic patients [29].

A personalized written action plan, meaning a document explaining how to deal with the condition, particularly in the event of an attack, and when to refer to the doctor or the emergency services, is widely accepted as good practice in asthma education and self-management [30]. According to the National Institute for Health and Care Excellence, providing patients with such a plan is a quality indicator and can improve outcomes such as self-efficacy, knowledge, and confidence for people with asthma. In those who have had a recent exacerbation, resulting in admission to hospital, it may reduce readmission rates [31]. Quite recently, a survey was conducted among 277 pediatricians in 6 European countries to assess the care for asthmatic children in the outpatients' setting in terms of therapeutic education; it turned out that 80% of the sampled patients received a personalized action plan during the visit (83% in Italy) [32]. Several papers consider the availability of an individualized action plan among the interventions that can help reducing ED visits for acute asthma [2, 20, 21]. Some authors also suggest the use of electronic action plans that might be delivered to the patients at ED discharge [33].

*Limitations of the Study*. This is a retrospective, single-center study which considers only one year of ED visits for asthma. Other settings, such as rural, non-Pediatric EDs, or community, and general practitioners' outpatient services are thus not included. We could not analyze the therapy performed and/or proposed at discharge, nor asthma severity which had not been evaluated or recorded during the ED visit. All children of any age were included; however, in those aged ≤12 months bronchiolitis might be mistaken for a wheezing bronchitis.

## 5. Conclusions

Despite the several limitations, our paper points out some important differences in asthma exacerbations between preschool- and school-aged children concerning etiology and seasonal trends, specific for our climate. It also highlights the need for an improved management of asthmatic patients outside the ED and a tighter connection with outpatients' services: individualized written action plans should always be available; moreover, recommending vaccinations and allergen-avoidance measures might be useful, as well as prescribing controller medications at ED discharge in those patients with known chronic asthma and under no therapy.

## Figures and Tables

**Table 1:** Classification of asthma severity according to the guidelines of the Italian Society of Pediatrics [8]. Normal values for respiratory rate: <2 months: <60 apm; 2–12 months: <50 apm; 1–5 yrs: <40 apm; 6–9 years: <30 apm; 10–14 yrs: <20 apm. Normal values for heart rate: 0–12 months: <160 bpm; 1-2 yrs: <120 bpm; 2–8 yrs: <110 bpm. Not all signs are needed for defining the severity of an exacerbation. apm: acts per minute; bpm: beats per minute; PEF: peak expiratory flow; FEV_1_: forced expiratory volume in the 1st second; SaO_2_: arterial oxygen saturation; PaCO_2_: partial pressure of carbon dioxide in arterial blood.

	Mild	Moderate	Severe	Life-threatening
Talk	Able to converse	Phrases	Words	None
Respiratory rate	Normal	Increased	Increased	Bradypnea/gasping
Colour	Normal	Pale	Pale/cyanosis	Cyanosis
Level of consciousness	Normal	Agitation	Agitation	Confusion/drowsiness
Wheezing	End of expiration	Expiration	Expiration/inspiration	Absent
Use of accessory muscles of respiration	Absent	Moderate	Remarkable	Paradoxical breathing
Heart rate	Normal	Increased	Increased	Increased/bradycardia
PEF-FEV_1_ (% of predicted or personal best)	>80%	60–80%	<60%	Nonexecutable
SpO_2_ (room air)	>95%	92–95%	<92%	<90%
PaCO_2_ (mmHg)	<38	38–42	>42	>42

**Table 2:** Characteristics of the 603 patients aged 0–14 years who were visited for acute asthma from 1 January to 31 December 2016 in the Pediatric ED of S. Orsola-Malpighi University Hospital of Bologna.

	Total (*n* = 603)	Aged <6 years (*n* = 459)	Aged ≥6 years (*n* = 144)
Males, *n* (%)	394 (65)	292 (64)	102 (71)
Age, yrs			
(i) Median	3.1	2.1	8.7
(ii) 25th percentile	1.5	1.3	6.9
(iii) 75th percentile	5.8	3.7	10.7
Etiology, *n* (%)			
(i) Infection	515 (85)	435 (95)	80 (56)
(ii) Allergy	60 (10)	12 (3)	48 (33)
(iii) Exercise	2 (0.3)	1 (0.2)	1 (0.7)
(iv) Unknown	26 (4.3)	11 (2)	15 (10)
Previous diagnosis of asthma/wheezing bronchitis, *n* (%)	191 (31)	96 (21)	95 (66)
Controller therapy, *n* (%)	61 (10)	27 (6)	32 (22)
Triage code, *n* (%)			
(i) White	94 (15.6)	61 (13.3)	33 (22.9)
(ii) Green	282 (46.8)	210 (45.8)	72 (50)
(iii) Yellow	225 (37.3)	186 (40.5)	39 (27.1)
(iv) Red	2 (0.3)	2 (0.4)	0 (0)
Severity of the exacerbation, *n* (%)			
(i) Mild	340 (56.4)	244 (53)	95 (66)
(ii) Moderate	237 (39.3)	194 (42)	43 (30)
(iii) Severe	26 (4.3)	21 (5)	3 (2)
(iv) Life-threatening	0	0	0
